# Risk factors of hepatitis B virus infection in Turkey: A population-based, case-control study: Risk Factors for HBV Infection

**Published:** 2011-04-01

**Authors:** Ali Ozer, Yusuf Yakupogullari, Ali Beytur, Leyla Beytur, Mehmet Koroglu, Feyza salman, Fisun Aydogan

**Affiliations:** 1 Beydagi State Hospital, Public Health Department, Malatya, Turkey; 2 Inonu University Medical School, Clinic Microbiology Department, Malatya, Turkey; 3 Inonu University Medical School, Urology Department, Malatya, Turkey; 4 Beydagi State Hospital, Obstetrics and Gynaecology Department, Malatya, Turkey; 5 Malatya State Hospital, Clinic Microbiology Department, Malatya, Turkey; 6 Malatya State Hospital, Microbiology Laboratory, Malatya, Turkey; 7 Malatya State Hospital, Infectious Diseases Department, Malatya, Turkey

**Keywords:** Hepatitis B virus, Transmission, Risk factor.

## Abstract

**Background:**

Although the World Health Organization (WHO) classifies Turkey as a country with a moderate-high prevalence of hepatitis B virus (HBV) infection, there is little data on HBV transmission in this country.

**Objectives:**

To identify risk factors for HBV infection, we performed a retrospective case-control study between January 2007 and December 2009.

**Patients and Methods:**

Acute HBV patients and population controls were selected, and data from these groups were analyzed by logistic regression method.

**Results:**

The study included 129 patients with acute HBV infection and 219 controls. Hemodialysis (OR:8.2, 95% CI: 4.17-16.61, p < 0.05), having an HBsAg (+) spouse (OR: 4.3, 95% CI:2.17-8.53, p < 0.05), living with an HBsAg (+) parent(s) (OR: 3.25, 95% CI:1.73-6.12, p < 0.05), and being male (OR: 1.34, 95% CI: 0.82-2.21, p < 0.05) were independent risk factors that were potentially associated with HBV infection. More than one-third of female patients had a significantly higher risk (34.5% vs. 13.5%, p < 0.05) of acquiring HBV from their sexual partners. Hemodialysis was the most frequent risk factor (46.9% vs. 20%, x (2) = 10.45, p < 0.05) for patients aged over 31 years, and living with HBsAg (+) parents was a significantly higher risk factor (28.8% vs. 10.2%, x (2) = 6.15, p < 0.05) that is more likely to lead to HBVin patients aged under 30 years.

**Conclusions:**

This study suggests that persons in Turkey who undergo hemodialysis are at high risk for acquiring HBV. Having an HBsAg (+) spouse (sexual transmission) or living with HBsAg (+) parents (household transmission) are significant risk factors for HBV transmission. Vaccination appears to be better preventive method against the spread of HBV.

## Background

Hepatitis B virus (HBV) is a leading cause of liver disease, affecting many populations throughout the world. Approximately one-third of living persons have been infected with HBV at one point in their lives, and 350 million of them have become chronic HBV patients or carriers. More than 1 million people die each year due to HBV-related diseases, such as chronic active hepatitis, cirrhosis, and hepatocellular carcinoma [1][2]. HBV is transmitted through several routes: drug use-related injections; exposure to HBV-positive blood or other body fluids; heterosexual or male homosexual sexual activity; from mother to infant (or vertically); and between children in a household (or horizontally)[3]. Identification of transmission routes can help improve preventive measures.

In Turkey, safe and effective vaccines have been available since the early 1990s, and a nationwide HBV vaccination program has been implemented since 1998. However, there are 4-4.6 million people with chronic HBV infection in Turkey [4]. The Health Ministry has reported that a total of 6600 new HBV infections have been diagnosed, 4 of whom died due to acute liver dysfunction in 2006[5]. Although Turkey is a country with an intermediate-high prevalence of HBV infection [6], the means by which HBV is spread in Turkey must be clarified.

## Objectives

In this study, we conducted a population-based, case-controlled study to determine the potential risk factors for HBV transmission in the Turkish population.

## Materials And Methods

### Study Design and Population

This study was conducted between January 2007 and December 2009 in Malatya City. This city is located in mid-eastern Anatolia and has an estimated urban population of 555,000 and an estimated rural population of 15,000. The study was performed in the Public Health Office with the participation of two state hospitals. To identify risk factors for HBV acquisition, we conducted a case-control study. The Public Health Department generated the case and control groups. All patients who were diagnosed with acute HBV infection within the study periods were eligible for inclusion. The case definition of acute HBV infection was made, with some modifications, using criteria per the Council of State and Territorial Epidemiologists (CSTE) [7]. Control groups consisted of healthy residents who met the demographic characteristics of the study population and who could be documented as negative for any current and/or previous HBV infection. Chronic HBV patients, vaccinated persons, healthy carriers, and formerly infected individuals were not included. Patients and controls were requested to provide written informed consent. All adult patients and controls were volunteers. Nonadult patients were included only with their parents' permission.

### Data collection

A wide range of possible risk factors were investigated with regard to HBV transmission [8]. All subjects were questioned about dental visits, barber visits, blood transfusions, circumcision, hospitalizations, having an HBsAg-positive spouse (wife, husband, or regular sexual partner), commercial sex use, commercial sex work, piercings, tattoos, intravenous drug use, narcotic addiction, homosexual contact, living with an HBsAg-positive parent, and working as a health care worker (HCW).

In interviews with the patients and controls, a standard questionnaire form that included these data was completed. The questionnaire had two formats, depending on the age of the respondent. One form was designated for nonadult patients (aged = 16 years), who consequently were not queried on sexual activity. Respondents were asked about their contact with potential risk factors within the last 6 weeks and within the last 6 months, which reflects the incubation period for HBV infection. The demographics of the cases and control patients, such as age, gender, marital status, educational level, and occupation, were recorded. The data nonadult patients were obtained with their parents' guidance.

Regarding the frequency of procedures, dental visits were coded into three categories: no dental visits were coded as negative, having made only one visit was coded as 1, and having made two or more visits within the incubation period was coded as 2. Visits for noninvasive procedures were excluded. Visits to the barber (or beauty salon) also included shaves (for males), manicures, pedicures, epilating (electrolysis), peeling, skin care, and acupuncture. Barber visits were also scored from 0 to ≥ 3, according to the frequency of the procedure in the previous 6 weeks and 6 months. Piercings included persons who experienced an insertion of any external accessory, such as earrings or ornaments, into the skin or mucosal tissue.

### Laboratory analysis

Serum samples were analyzed for HBV markers using EIA kits from Abboth-Axymys (US) and Roche-Cobas 6000 (US), in the clinical microbiology laboratories at both participating hospitals. HBsAg, anti-HBs, anti-HBc-IgG and IgM, HBeAg, and anti-HBe were measured for each patient. Biochemical parameters, such as AST, ALT, and ALP, were also measured using the Abboth System (US) and Beckman-Coulter (US) systems simultaneously.

### Statistical analysis

We used the Statistical Package for Social Sciences (SPSS Inc. Chicago, IL, USA), version 11.0 for Windows, to perform the statistical analyses. The data were initially tested for normal distribution by Kolmogorov-Smirnov test and found to be normal (p > 0.05). We used the multiple logistic regression and chi-square tests for statistical analysis. (P < 0.05) was accepted as statistically significant.

## Results

### Patients and controls

**Figure 1:**
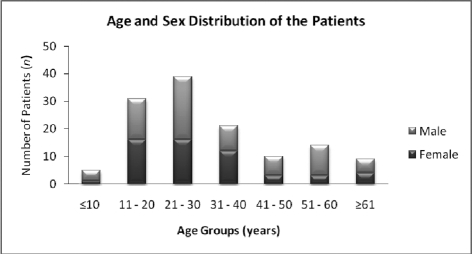
Age and sex distribution of patients with acute HBV infection

During the study period, a total of 155 patients were diagnosed with acute HBV infection, 138 of whom were willing to participate in the study. The remaining 17 patients were excluded (12 refused to be included, and 5 were unavailable for interview). In addition, 9 patients were excluded due to incomplete data, as they failed to complete the questionnaire forms. The mean age of the 129 participating patients was 30.4 ± 17.5 years (ranging from 7 to 80 years). The study group was predominantly male (57.4%/42.6%). The age and sex of the patients are shown in Figure 1 . Thirty-nine cases were hemodialysis patients, and the remaining 90 patients were healthy individuals.

Nearly half of the patients (48.1%) earned over 1600 Turkish Liras (1200 USD) per month per family, which is the average income for a Turkish individual. Nineteen percent of the patients declared that they were university graduates, and 56.6% said that they graduated from high school. Seventy-one percent were residents of Malatya City, and 27% came from the surrounding rural areas. Of the 410 individuals who were evaluated, 219 were eligible for inclusion as control subjects. The mean age of the controls was 30.8 ± 17.5 years (ranging from 7 to 80 years), and the male/female ratio was 66.2%/33.8%. Both groups shared similar socioeconomic and demographic characteristics.

### Risk factors

Thirty-nine patients reported hemodialysis, 35 reported over two dental visits, 25 reported over three barber visits, 29 reported having an HBV-positive spouse, 28 reported living with an HBsAg-positive parent, 11 reported hospital stays, 3 reported blood transfusions (all were multiple transfusions), 4 reported piercings (3 were earrings, 1 was a nasal piercing), 2 reported circumcisions, 2 reported multiple sex partners, and 1 reported commercial sex use. Nine (6.9%) patients did not select any potential risk factor. No patient declared homosexual activity, having tattoos, narcotic use, intravenous drug use, commercial sex work, or an HCW job. By logistic regression analysis of the two groups, hemodialysis, having an HBV-positive spouse, living with HBsAg-positive parent, and male gender were found to be independent risk factors for acquisition of HBV. Table 1 shows the results of the multivariate analysis for HBV infection.

When the identified risk factors were analyzed, females had a significantly higher risk for acquiring the virus from their positive spouse. However, males experienced no such pattern. Living with an HBsAg-positive parent and undergoing hemodialysis were significant risk factors for patients aged under 30 and over 31 years, respectively. Table 2 and Table 3 show the statistical analysis of the risk factors, based on patient age and gender.

**Table 1:** Multivariate analysis of factors potentially associated with acute HBV infection

**Factors**	**Patients **No. (%)	**Controls **No. (%)	**Odds ratio**	**95% CI**	**p-value**
**Gender**					
**Female**	55 (42.6)	74 (33.8)	1.00	0.82-2.21	0.245
**Male**	74 (57.4)	145 (66.2)	1.34		
**Hemodialysis**					
**Positive**	39 (30.2)	14 (6.4)	8.32	4.17-16.61	< 0.001
**Negative**	90 (69.8)	205 (93.6)	1.00		
**HBsAg (+) Spouse**					
**Positive**	29 (22.5)	18 (8.2)	4.30	2.17-8.53	< 0.001
**Negative**	100 (77.5)	201 (91.8)	1.00		
**HBsAg (+) Parents**					
**Positive**	28 (21.7)	26 (11.9)	3.25	1.73-6.12	< 0.001
**Negative**	101 (78.3)	193 (88.1)	1.00		

**Table 2:** Distribution of the risk factors according to gender

** Risk Factors **	** Gender **		** x (2) **	** p-value **
	** Male **No. (%)	** Female **No. (%)		
** HBsAg (+) Spouse **	10 (13.5)	19 (34.5)	8.00	< 0.001
** Hemodialysis (+) **	24 (32.5)	15 (27.3)	0.39	0.52
** HBsAg (+) Parents **	20 (27.0)	8 (14.5)	2.89	0.09

**Table 3:** Distribution of the risk factors according to age

**Risk Factors**	**Age **(years)		**x(2)**	**p-value**
	**≤ 30**No. (%)	**≥ 31 **No. (%)		
**HBsA (+) Spouse**	15/18.8	14/28.6	1.68	0.20
**Hemodialysis (+)**	16/20.0	23/46.9	10.45	< 0.001
**HBsAg (+) Parents**	23/28.8	5/10.2	6.15	0.01

## Discussion

The public health burden of HBV infection is due almost entirely to its long-term effects on liver function. In addition to health problems, these diseases cause broader social and economic problems. More than $1 billion is spent annually for hepatitis B-related hospitalizations [9]. Although it is very difficult to measure, the total cost of HBV might be several magnitudes greater.

HBV is acquired by percutaneous and mucosal exposure to the body fluids of an infected person. However, there is considerable variation in transmission mode between geographic areas and populations [2]. In endemic countries (≥ 8% chronic HBV patients), most infections are acquired prenatally or during childhood via close household contacts [10]. Alternatively, in countries where the prevalence of HBV is low (≤ 2 chronic HBV patients), most infections are acquired in adult life [11].

HBV positivity is associated with a history of multiple sex partners, male homosexual activity, and illicit drug use (IDU and/or intranasal cocaine use) in western countries [12][13]. Correspondingly, in addition to the predominance of sexual transmission in adults, Hahné et al. [14] reported that having parent(s) born in a highly endemic country is a significant risk factor in the acquisition of HBV among children in the Netherlands. Hutin and colleagues [15] emphasized nosocomial transmission, reporting that injections in health care settings are a major source of HBV transmission in Moldova, a result of the widespread reuse of inadequately sterilized single-use syringes.

In this study, we determined that hemodialysis, marriage to an HBsAg-positive person, and living with HBsAg-positive parent(s) were independent risk factors for HBV transmission in a Turkish population (Table 1). Although the frequency of other proposed risk factors, such as dental visits, barber visits, blood transfusions, and surgery, was higher in the patient group, no significant difference was observed in the controls. No patient reported homosexual activity, tattoos, narcotic use, intravenous drug use, or commercial sex work, suggesting that direct transmission through these routes is infrequent in Turkey. Nonetheless, these activities are predominant behaviors that are associated with HBV patients in many western countries. This difference might be a reflection of the sociocultural characteristics of the Turkish population. Nevertheless, it is possible that some of our subjects might have been reluctant to declare such activities due to social concerns.

More than half of our patients were young adults (age range, 15-40 years) (Figure 1). This result may be due to the rising incidence of risk factors toward the end of adolescence. In contrast, we noted a decrease in the number of patients aged in their 40s and a slight increase in those over 50 years (accounted for primarily by hemodialysis patients) (Figure 1). When independent risk factors were analyzed with respect to age and gender, living with HBsAg-positive parent(s) was a significantly predominant risk factor among patients aged under 30 years. We believed that this was most likely due to household transmission (Table 2). Furthermore, hemodialysis was a significant risk factor for patients aged over 31 years (Table 3), possibly due to the increasing incidence of chronic renal diseases with age in our population.

In 2003, The Health Ministry reported that the proportion of patients aged 0-9 years accounted for 6% of acute HBV patients [16]. However, we found few cases among children aged under 10 years (n = 5, 3.8%) throughout the study period. We believed that this is a result attributed to the national vaccination program. Parents of these pediatric patients reported that they could not continue the vaccination program after the initial dose. In addition, no protective antibody titer was detected in these five children. Not surprisingly, in 2006, official statistics suggested that noncompliance with HBV-3 vaccination at age 0 ranged from 11% to 20% across the country [5].

We found that hemodialysis was the most significant risk factor for HBV transmission in our study. However, hemodialysis patients in Turkey are conventionally vaccinated against HBV. Additionally, HBV-positive-labeled devices are not used for negative patients in large centers. The poor condition of facilities with dialysis units (particularly in private health care centers), the few devices that are available to large populations, and the low adherence to infection control measures (i.e., inadequate disinfection of devices by patients) might be some of the major reasons for the significant device-associated HBV transmission in Turkey.

Furthermore, a dialysis patient undergoes several rounds of dialysis until vaccinations are able to provide a protective antibody titer. Although it is rare, occult HBV infections among patients with chronic renal failure risk can contaminating HBV-negative-labeled devices [17]. When other risk-bearing activities, such as blood transfusions and surgery, were pooled with hemodialysis, nosocomial transmission of the pathogen was identified as one of the most significant transmission modes in the studied population our population. Therefore, our results suggest that nosocomial hygiene and infection control measures must be improved to reduce HBV transmission in Turkey.

A total of 29 patients reported having an HBsAg-positive sexual partner. Our statistical analysis revealed a significant predominance of this risk factor for women compared with male patients (13.5% vs. 34.5, p < 0.05) (Table 3). When hemodialysis patients (n = 15) and sexually inactive patients (n = 9) were excluded from the female cases, 61.2% of the women were likely to have acquired the virus from their positive partners. In fact, a number of laws that are designed to prevent the spread of communicable diseases in Turkey have been written over the past 10 years. For example, couples are asked undergo a number of blood tests (e.g., HBsAg, anti-HIV-I/II, VDRL, and anti-HCV) when they apply to be married. Anyone who is found to be HBsAg-positive in such tests is directed to an infectious diseases resident to learn about the disease and infection control measures (vaccinations, condom use, etc).

However, these activities do not provide adequate prevention against HBV transmission. For example, although a spouse is vaccinated, couples can marry before protective immunity is established. In addition, in Turkey, due to its large traditional population and the strict male dominance of social life, women often face considerable obstacles in following a regular vaccination program.

Interestingly, two young female patients whose husbands were HBsAg-positive reported that they had refused to be vaccinated because they were worried that the vaccinations could cause them to lose their reproductive capacities before getting married. A low educational level and superstitious populations might be the chief reasons for such problems. Additionally, many female patients reported that their HBV-positive husbands had simply chosen not to use condom. We believe that such characteristics of our cultural structure increase the risk of HBV transmission from males to females in our country.

In Turkey, Kose et al. [18] recently reported that HBV transmission in 958 chronic HBV patients was associated with having an HBV (+) family member (80.6%), being HCW (9.2%), hemodialysis (2.8%), and being an oncology patient (2.1%). However, we selected only acute HBV patients in this study. We believe that our method is likely to provide more accurate and current data, in contrast to chronic HBV patients with long histories.

As there has been no successful treatment option for chronic patients, the principal strategy for controlling HBV infection is vaccination. The World Health Organization has recommended routine HBV vaccinations for newborns since 1992. In Turkey, recombinant HBV vaccine is available at primary care centers free of charge to any applicant, and a nationwide vaccination program has been in existence for the past 11 years. This program entails the immunzation of all children at birth, vaccinations and immunoglobulin injections to the newborn if the mother is HBsAg-positive, and routine immunization of high-risk people (e.g., HCWs).

In the United States, between 1990 and 2002, the incidence of acute HBV infection declined from 8.5 to 2.8 (per 100,000) in the general population after the implementation of a routine HBV vaccination program. However, the most significant decline in acute hepatitis B incidence occurred among persons aged 0-19 years, from 3.0 in 1990 to 0.3 in 2002 [19]. In contrast, the Turkish Health Ministry reported that the incidence of the disease rose unexpectedly from 4.55 to 10.05 (per 100,000 of the population) between 1990 and 2006 [5], the reasons for which have not been identified. We believe that despite the vaccination program, the increased sophistication of medical recording and reporting systems and the rising standards for assessments have merely clarified the actual status of HBV in the country.

The World Health Organization categorizes HBV infection as an occupational disease for certain groups. For 13 years, the Health Ministry has regulated HCWs for HBV vaccination and have recommended that they ensure that protective immunity has been provided. In this study, we did not identify any HCW with an acute HBV infection. We believe that the highest compliance rates of HCWs with HBV vaccination programs are most likely due to their exposure to recurrent informational activities about the disease.

The results of this study suggest that transmission of HBV takes place predominantly via nosocomial routes in older patients and by sexual and household routes in younger patients in Turkey. Such investigations are important for the identification of potential risk factors and the determination of high-risk groups and behaviours. Although the more than 10 years of the vaccination program has led to a remarkable decrease in the incidence of HBV infection among children and certain groups (such as HCWs), it is still not possible to claim that reliable protection has been achieved for the Turkish society. Education of the public about the disease may reduce sociocultural resistance to preventive measures including vaccination activities. Providing a protective antibody titer in the early years of life may protect individuals against HBV acquisition, as will be determined soon.
